# Rapid recovery of locomotor performance after leg loss in harvestmen

**DOI:** 10.1038/s41598-020-70557-2

**Published:** 2020-08-13

**Authors:** Ignacio Escalante, Marc A. Badger, Damian O. Elias

**Affiliations:** 1 Department of Environmental Sciences, Policy, and Management, University of California - Berkeley, Berkeley, CA 94720 USA; 2 Department of Integrative Biology, University of California - Berkeley, Berkeley, CA 94720 USA; 3 Present Address: Department of Computer and Information Science, University of Pennsylvania, Philadelphia, PA 19104 USA

**Keywords:** Animal behaviour, Biomechanics, Entomology, Behavioural ecology, Evolutionary ecology, Tropical ecology

## Abstract

Animals have evolved adaptations to deal with environmental challenges. For instance, voluntarily releasing appendages (autotomy) to escape potential predators. Although it may enhance immediate survival, this self-imposed bodily damage may convey long-term consequences. Hence, compensatory strategies for this type of damage might exist. We experimentally induced autotomy in *Prionostemma* harvestmen. These arachnids are ideal to examine this topic because they show high levels of leg loss in the field but do not regenerate their legs. We video-recorded animals moving on a horizontal track and reconstructed their 3D trajectories with custom software tools to measure locomotor performance. Individuals that lost either three legs total or two legs on the same side of the body showed an immediate and substantial decrease in velocity and acceleration. Surprisingly, harvestmen recovered initial performance after 2 days. This is the quickest locomotor recovery recorded for autotomizing animals. We also found post-autotomy changes in stride and postural kinematics, suggesting a role for kinematic adjustments in recovery. Additionally, following leg loss, some animals changed the gaits used during escape maneuvers, and/or recruited the ‘sensory’ legs for locomotion. Together, these findings suggest that harvestmen are mechanically robust to the bodily damage imposed by leg loss.

## Introduction

Animals face a myriad of challenges during their lives, including predation, parasitism, navigating obstacles, and physiological stress. These challenges often lead to damage and many animals have evolved adaptations to compensate for these injuries. Compensation for damage often involves gradual improvements using developmental, morphological, or behavioral changes^1,2^. For instance, animals such as lizards, crickets and damselfly larvae reduce their mobility and become more cryptic after damage from potential predators^3–6^.

While bodily damage is often unintended, in some species injury is self-imposed and potentially adaptive. For example, many animals voluntarily lose appendages when grabbed by potential predators, a defensive strategy known as autotomy^7^. Although important for immediate survival^5,7–10^, the loss of body parts may compromise other aspects of organismal function and, by extension, an individual’s long term fitness^11,12^. Effects of autotomy include changes for locomotion, foraging, development, sensory biology, longevity, migration, and survival^13–15^.

With regards to locomotion, stability and maneuverability are altered by autotomy, as found for green anole lizards^16^ and leopard geckos^17^. Locomotor performance (i.e. acceleration or velocity), often interpreted as evidence of the ability to escape a potentially dangerous interaction, is also affected by autotomy^7^. Accordingly, wolf spiders missing legs are slower than intact individuals when running^18,19^. Besides performance metrics, another set of movement parameters (stride and posture kinematics) can change after autotomy. For instance, stride length and duty factor (the proportion of time during a stride that each leg is on the substrate instead of the air) changed in cellar spiders when walking on inclines^20^. Forelimb stance width and limb posture changed in *Anolis* lizards when running on narrow surfaces^16^. Additionally, the overall timing of all the legs used during locomotion (gaits) by arthropods has provided important insights on the effects of leg loss. Changes in the timing of using specific legs, as well as the multi-legged coordination to modify tetrapod or tripod gaits after autotomy has been recorded in tarantulas^21^, cockroaches^22,23^, crabs^24^, and ants^25^.

Given these negative impacts of autotomy on locomotion, a critical question is whether animals can compensate for this. Monitoring post-autotomy locomotor performance over time generates insights into the mechanisms for compensation and recovery. For example, in leopard geckos, body posture during locomotion changed immediately after tail autotomy but recovered to initial levels in 2–10 weeks, matching the time course for tail regeneration^26^. In contrast, tail autotomy in skinks caused a decrease in sprint speed, which did not recover after 4 weeks, even though endurance recovered over that timeframe^27^. Finally, among *Anolis* lizards, some individuals recovered in-air stability over the course of five weeks post tail autotomy^28^. Together, these findings suggest that some animals can recover locomotor performance over time. Additionally, wolf spiders undergo postural and kinematic adjustments aimed to maintain stability while moving after losing legs^29^. Some spiders changed their gait to a modified tripod gait or moved with an ablated tetrapod gait^29^. To maintain speed and stability after autotomy, spiders made small spatial changes in each leg position, as well as decreases in the proportion of time during a stride in which only two legs were on the ground as opposed to four^29^.

Here, we tested for the long-term consequences of autotomy of locomotion, as well as the potential kinematic and behavioral strategies harvestmen can use to mitigate the effect of bodily damage. This group of arachnids is ideal for exploring this topic because natural levels of leg autotomy are high, ranging between 33 and 58% of individuals in a population^30–33^. Additionally, harvestmen do not regenerate their legs even if lost before maturity^34^, contrary to other arthropods that regenerate legs after molting.

Despite having eight legs, harvestmen move using six legs in an alternate-tripod gait^35,36^, similar to terrestrial insects^36–39^. Legs of the second pair serve a sensory function^40^. Harvestmen use up to four different gaits to move (running, stotting, bobbing and walking), each of which differs in their kinematics, trajectory, and gait diagrams^35^. Previous work has shown that harvestmen missing legs experience immediate decreases in speed that are attributable to substrate properties and the number of legs lost^32,33^. To date, however, recovery from leg loss has not been examined, nor have the kinematic mechanisms associated with recovery.

The goal of this study was to experimentally test the hypothesis that autotomy affects locomotor performance, from which *Prionostemma* harvestmen can recover. We predicted that (1) locomotor performance would decline immediately after autotomy, but (2) harvestmen would recover performance over time, which would correlate with kinematic adjustments. Additionally, we expected that (3) negative consequences of leg loss on velocity and acceleration would be greater for animals missing more legs, missing locomotor (versus sensory) legs, and missing legs on the same (versus opposite) sides of the body. Finally, we predicted (4) changes in the gait performed and/or the legs used during locomotion. Altogether, we expected harvestmen to be robust to bodily damage, defined in this case as the ability to withstand and overcome the negative consequences of autotomy on locomotion.

## Materials and methods

### Study animals

Research occurred in the Neotropical lowland rainforest at La Selva Biological Station, Costa Rica (10° 26′ N, 84° 00′ W, 50 m elevation), from June to August 2015. We studied a currently undescribed species of *Prionostemma* Pocock harvestmen (Opiliones: Sclerosomatidae, referred to as *P.* sp1. in Refs.^35,41–43^, which roosts during daytime in tree trunks, buttresses, and palm leaves^44^. At nighttime, they actively forage on the ground and foliage^41,42^. Harvestmen face a wide diversity of predators, including small mammals, lizards, frogs, spiders, centipedes, and insects^45^. We collected 135 eight-legged individuals and placed them in clear plastic deli containers (15 × 12 × 10 cm) 24 h before trials. Individuals were fed cucumber, apple, and cat food in captivity every 2 days.

### Experimental setup

We recorded harvestmen moving during daytime across a horizontal arena (Supplementary Video S1) in the lab, using the same procedure as Ref.^35^. To simulate predation attempts, we grabbed individuals by their hind legs and then released them. Hence, we interpreted the animal’s subsequent movements as escape behaviors^46^. Trials were video recorded with a GoPro HERO4 camera (GoPro, San Mateo, CA, USA) at 120 frames/s. A mirror at 45° perpendicular to the ground on the opposite side of the arena to the camera allowed recording lateral and dorsal views.

The accurate 3D body’s location in the videos was obtained as follows. The focal length, optical center, and radial and tangential lens distortions (i.e. intrinsic camera parameters) for the GoPro camera and lens were obtained using the built-in checkerboard calibration app in MATLAB vR2016a (The Mathworks, Natick, MA, USA). Then, the translation and orientation of the lateral and dorsal views relative to the track (i.e. the extrinsic camera parameters) were estimated using an M-estimator sample consensus algorithm.

### Experimental treatments and trials

We induced autotomy by gently grasping the femur of the target leg with forceps as Refs.^20,31,33^, which immediately resulted in the release of that leg at the coxa-trochanter joint. We measured locomotion for each animal at seven different times: once prior to autotomy, once immediately after, and then 2 h, 6 h, 24 h, and 2 days after autotomy.

**Figure 1:**
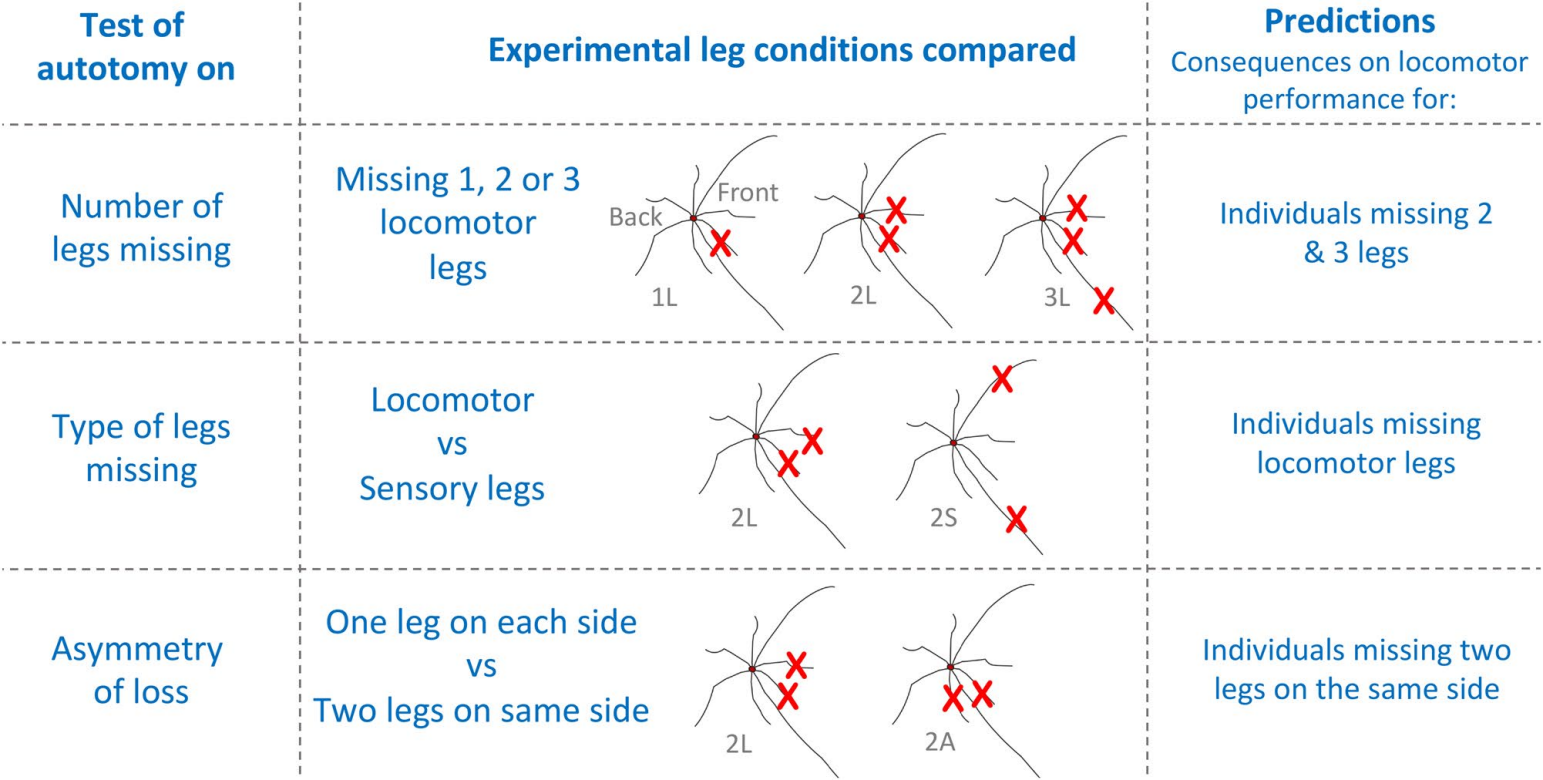
Experimental comparisons. Body diagrams in the center column represent *Prionostemma* sp.1 harvestmen seen in dorsal view. The main prediction for each comparison is described in the right column. Figure was created in Microsoft Power Point version 16.39 (URL: https://www.microsoft.com/en-us/microsoft-365/microsoft-office).

The experimental treatments used here varied in the number and type of legs missing (Fig. 1) to reflect the natural occurrence of autotomy. The treatments are named with a number representing the number of legs lost, and a letter representing the type of loss. Treatments were (1) C: intact control individuals with 8 legs. Legs were grabbed with forceps but without inducing autotomy, (2) 1L: missing the right locomotor leg I, (3) 2L: missing both of the locomotor legs I, (4) 2S: missing both sensory legs (legs II), (5) 2A: asymmetrical loss, missing legs I and III from the same side, and (6) 3L: missing three legs (both locomotor legs I and one leg II). Individuals were randomly assigned one treatment (n = 21–24 per treatment). We induced autotomy of hind legs, given that our field survey showed that missing hind legs was 53% more likely than missing rear legs.

### Video analyses

We digitized videos using the custom scripts developed in Mathematica 10.4 (Wolfram Research, Inc., Champaign, IL, USA) described in Ref.^35^. Briefly, scripts automatically tracked the position of the animal’s body across each view. We then reconstructed its three-dimensional trajectory over time using built-in functions (i.e. estimateFundamentalMatrix and triangulate) and tools developed by Ref.^47^ for MATLAB. Using the XYZ trajectory of body position, we calculated the kinematics of the animal’s center of mass (CoM). Automatically tracking the movement of each leg was not possible, unfortunately. Harvestmen legs are very thin (Supplementary Video S1), which prevents a good contrast with the background.

We calculated nine performance, postural, or stride variables to describe harvestmen locomotion as in Escalante et al.^35^. For performance metrics, we used the XYZ positions over time to calculate (1) the average horizontal velocity, hereafter referred to as ‘velocity’, calculated as v_h = (x_final − x_initial)/(t_final − t_initial), where x_initial and x_final are the (x, y) coordinates of the body at the start and end of the trial, respectively. (2) Maximal horizontal acceleration, calculated as a_hmax = max_{t \in trial} a_h(t), where a_h(t) is the horizontal component of the acceleration calculated from a quintic smoothing spline fit to 3D position over time. We consider these variables reflect biologically relevant performance. We assumed that harvestmen would aim to sustain fast speed to avoid being captured (velocity), as well as a fast burst of speed to quickly move away from a potential predator (acceleration).

For postural variables, we calculated (3) the three-dimensional sinuosity normalized by time. This unitless measurement is the total path length of the trajectory divided by the linear distance between the endpoints and quantifies the lateral and vertical deviations from a straight path^48^. We also measured (4) the minimal and (5) the maximal height of the CoM.

For stride kinematics, we visually followed the movement of a focal leg (left leg I). We noted the time when each leg was on the ground (stance phase), and when it was lifted (aerial phase), which together represent one stride. For 2L and 3L treatments we followed the third left leg as the focal leg. We followed three strides to calculate (6) the average duty factor, the proportion of time during each stride that the focal leg was on the ground, (7) average stride frequency, the number of complete strides per second, (8) average stride period, the time to complete one stride, and (9) average stride length, the maximal distance along the x-axis the leg moved during one stride.

To investigate patterns of leg use, we visually followed all legs during three strides and constructed gait diagrams. We did this for five individuals in each experimental treatment, before autotomy, immediately after, as well as 2 days after autotomy. Finally, we visually scored each video based on the type of gait performed. We grouped the type of gaits into “fast gaits” (running and stotting) and “slow gaits” (bobbing and walking). We grouped gaits this way because performance (velocity and acceleration) is similar within gait groups^35^. Additionally, our focus here was on understanding the consequences of autotomy regardless of gait type.

### Body measurements

We measured the length of the left leg IV for each individual to the nearest 0.05 mm using digital calipers. Leg IV length (see Supplementary Table S2) is a good proxy of body size since leg IV was never autotomized, and it correlated with leg I and III lengths (r = 0.39, 0.49, respectively. *P* < 0.02 for both. See Supplementary Fig. S1). Leg morphology is not sexually dimorphic^40^ and thus we included both adult females and males in this study. Voucher specimens of all individuals are preserved in 70% ethanol in the Essig Museum of Entomology, University of California Berkeley.

### Data analyses

To test the influence of autotomy on the locomotor performance we performed generalized linear mixed models (GLMMs) using velocity or acceleration as the response variable. Predictor variables included as fixed effects were treatment, time since autotomy (treated as categorical, hereafter referred to as ‘time’), gait group (fast or slow), and all possible interactions (Table 1). Individual identity was included as a random effect to account for repeated measurements. Preliminary analyses revealed that neither the leg length nor sex affected locomotor variables (GLM: leg length, sex, and leg length × sex interaction all *P* > 0.42). Hence, these variables were excluded from the final models. None of the nine kinematic variables were normally distributed (Shapiro tests *P* < 0.05). However, GLMMs and GLMs are robust to deviations from normality, so they were useful for between treatment and time comparisons.

**Table 1:** Results of likelihood ratio tests for the linear mixed models (GLMMs) to analyze the locomotor performance (velocity and acceleration) of *Prionostemma* sp.1 harvestmen over time.

Performance variable	Comparison	1					2					3				
		Number of legs					Type of legs					Side of body				
	Treatments included parameter	C-1L-2L-3L					C-2L-2S					C—2L—2A				
		AIC	Deviance	× 2	df	*P*	AIC	Deviance	× 2	df	*P*	AIC	Deviance	× 2	df	*P*
Average horizontal velocity (cm/s)	Complete model	4,040	3,923				3,150	3,063				3,193	3,105			
	Time	4,020	4,006	62.7	24	**0.00001**	3,153	3,141	58.4	18	**0.00001**	3,178	3,166	45.9	18	**0.0003**
	Treatment	4,016	3,997	53.5	21	**0.0001**	3,125	3,105	21.3	14	0.09	3,169	3,149	28.7	14	**0.01**
	Gait group	4,345	4,286	342.4	1	**0.00001**	3,369	3,323	239.5	1	**0.00001**	3,372	3,327	208.0	1	**0.00001**
	Treatment × time	4,002	3,977	33.4	18	**0.015**	3,127	3,103	19.1	12	0.09	3,167	3,144	24.0	12	**0.02**
	Treatment × gait	4,005	3,943	2.2	3	0.54	3,131	3,084	1.7	2	0.43	3,168	3,120	3.3	2	0.20
	Trail × gait	4,009	3,941	3.3	6	0.77	3,134	3,082	4.6	6	0.59	3,169	3,117	1.9	6	0.93
	Treatment × time × gait	4,017	3,937	14.0	18.0	0.74	3,141	3,077	14.3	12	0.28	3,179	3,115	10.6	12	0.56
Maximal horizontal acceleration (cm/s^2^)	Complete model	11,341	11,225				8,834	8,746				8,856	8,770			
	Time	11,293	11,279	17.6	6	**0.007**	8,807	8,796	9.8	6	0.13	8,831	8,819	17.5	8	**0.03**
	Treatment	11,290	11,270	8.7	3	**0.03**	8,815	8,795	9.1	2	**0.01**	8,838	8,818	16.0	4	**0.002**
	Gait group	11,351	11,327	65.7	1	**0.00001**	8,855	8,833	47.5	1	**0.00001**	8,869	8,847	45.9	3	**0.00001**
	Treatment × time	11,287	11,261	16.3	18	0.57	8,809	8,785	14.4	12	0.27	8,829	8,801	15.0	12	**0.04**
	Treatment × gait	11,307	11,245	2.4	3	0.49	8,819	8,871	3.6	2	0.17	8,842	8,794	6.7	2	0.26
	Trail × gait	11,311	11,243	6.3	6	0.39	8,820	8,767	9.8	6	0.13	8,839	8,787	5.6	6	0.47
	Treatment × time × gait	11,316	11,236	11.3	18	0.88	8,821	8,746	11.5	12	0.48	8,845	8,781	11.5	12	0.49

Treatment codes: numbers represent number of legs lost, and letters represent either the control group (C) or the type of leg condition (L—lost locomotor legs, S—lost sensory legs, and A—asymmetric loss, two legs on the same side of the body).

To test for each factor, a second GLMM without the variable of interest was compared with an ANOVA of the complete model. Significant difference between models at the *P* < 0.05 level are marked in bold. Individual identity was used a random factor in all models.

To identify the variables (and interactions) that affected locomotor performance we ran likelihood ratio tests^49^ for each model. Three sets of GLMMs (Fig. 1, Table 1) compared for the effect of (1) the number of missing legs (comparing C, 1L, 2L, and 3L treatments), (2) the type of missing leg (C, 2L and 2S), and (3) the side of the body where harvestmen lost legs (C, 2L, and 2A). Post hoc Tukey comparisons examined for differences within treatments over time, as well as differences between treatments at a given time after autotomy. We defined locomotor recovery as the first time point at which the mean locomotor performance was statistically indistinguishable from pre-autotomy levels following experimental leg loss.

Lastly, to examine potential changes in the gait use after autotomy, we compared the number of individuals in each treatment that performed each gait type before and immediately after autotomy using an independence chi-square. To examine gait type changes over time we compared the numbers before and two days after autotomy with an independence chi-square for each treatment. Statistical analyses were run in R (R Development Core Team 2018). A small portion of this dataset (the before autotomy trials of all individuals) were collected as part of another study describing gait kinematics^35^. Hence, information collected from some of the same individuals in Ref.^35^ were included in the current dataset, along with five other time points.

## Results

### Autotomy in the field

The levels of autotomy are high in this population. Of 399 surveyed individuals, 69% were missing at least one leg, 38% of animals were missing one leg, 23% missing two, 8% missing three, and 1% were missing four legs. We also observed that 2% of animals were missing the two sensory legs, 4% were missing one locomotor leg on each side of the body, and 3% were missing two locomotor legs on the same side of the body.

### Locomotor performance

#### Number of legs lost

**Figure 2:**
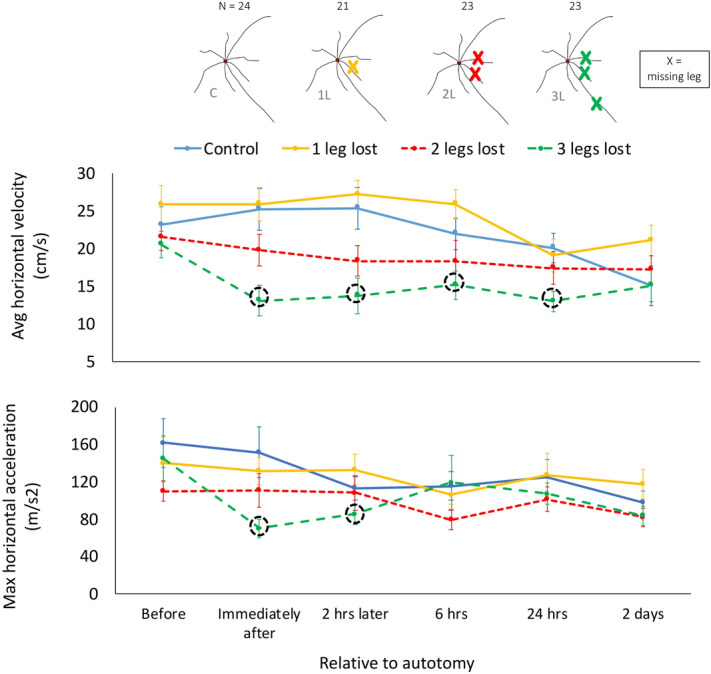
Locomotor performance over time for different levels of leg loss. Mean velocity (top) and acceleration (bottom) (± standard error) over time in *Prionostemma* sp.1 harvestmen with different levels of leg loss. Treatments here test for the effect of the number of legs lost. Dotted circles represent statistically significant differences from pre-autotomy levels of that treatment. Major effects of time and treatment are based on the GLMMs tests (Table 1) and the dotted circles reflect post hoc tests (see Supplementary Table S3). Graph shows all gaits pooled. Plot shows data up to 2 days after autotomy. Sample size (N) is included on the top of each treatment’s diagram. Figure was created in Microsoft Excel version 16.39 and Microsoft Power Point version 16.39 (URL: https://www.microsoft.com/en-us/microsoft-365/microsoft-office).

We found that velocity and acceleration differed between leg conditions and over time (GLMM: both factors *P* < 0.0001, Table 1, Supplementary Table S1). Post hoc comparisons revealed that locomotor performance did not differ among treatments before autotomy (*P* = 0.17, Fig. 2), but differed immediately after autotomy (*P* < 0.0001, Fig. 2). Specifically, animals missing three locomotor legs (3L treatment) decreased in both measures of performance (*P* < 0.05 for both comparisons). No changes were observed in the Control, 1L or 2L treatments (all tests *P* > 0.05). There was a significant treatment × time interaction (*P* = 0.02) in velocity, but not for acceleration (Table 1). Velocity decreased 40% on average and acceleration decreased 54% on average immediately after autotomy in 3L individuals (Fig. 2). However, animals in the 3L treatment recovered pre-autotomy velocity two days after leg loss (Fig. 2, Supplementary Video S1). At that point, velocity was indistinguishable from pre-autotomy levels (*P* > 0.05 for all Tukey tests) (Fig. 2, Supplementary Fig. S3 and Supplementary Tables S1, S3). Additionally, velocity was indistinguishable from pre-autotomy levels two days after autotomy in all treatments (see Supplementary Fig. S3). The recovery of acceleration occurred even earlier, 6 h after leg loss (Fig. 2). These findings suggest that harvestmen that lost three locomotor legs completely recovered locomotor performance two days after autotomy.

#### Type of missing legs

**Figure 3:**
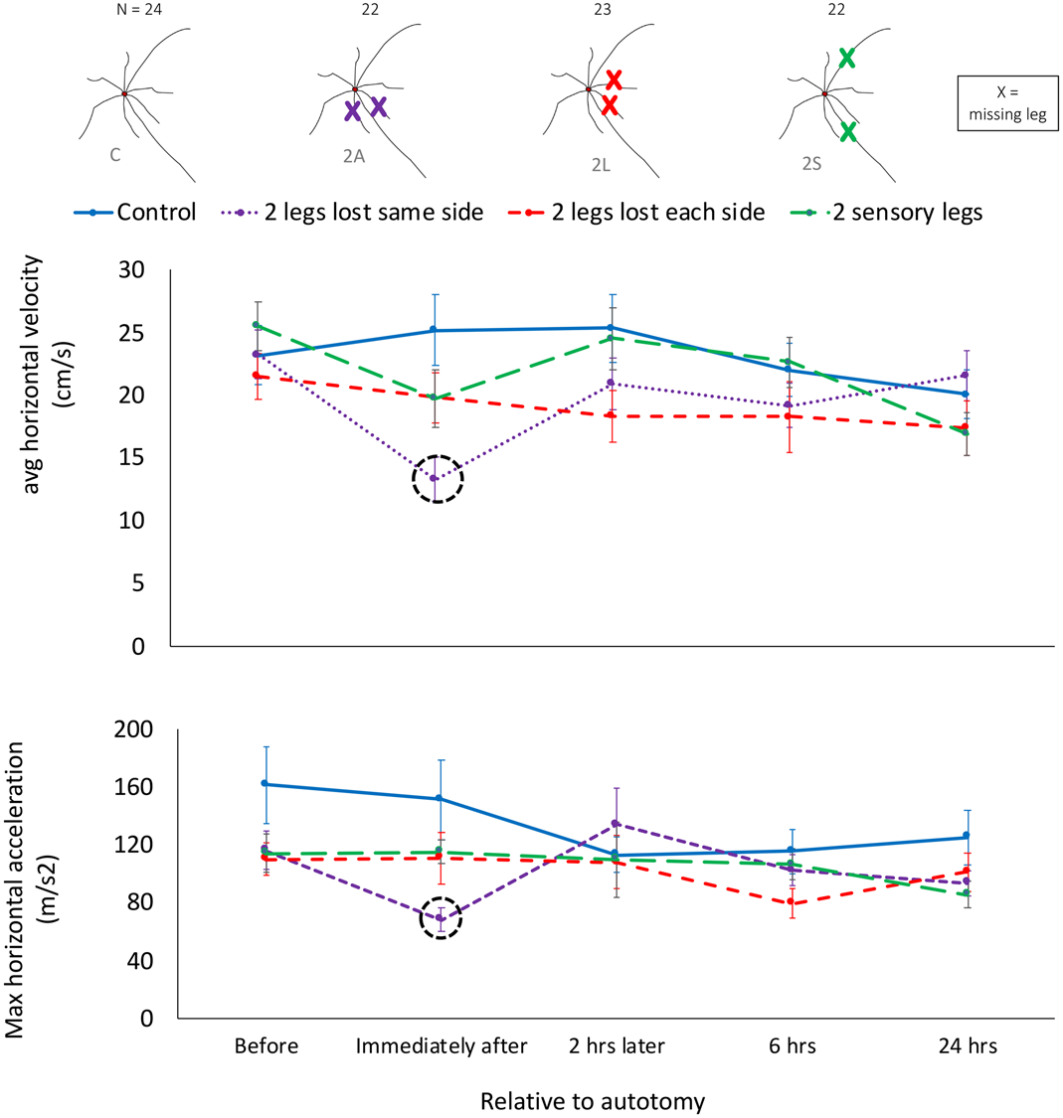
Locomotor performance over time for different combinations of leg loss and loss of different leg types. Mean velocity (top) and acceleration (bottom) (± standard error) for different patterns of leg loss in *Prionostemma* sp.1 harvestmen. Treatments here show the effects of (1) the type of leg missing (C-2L-2S), and (2) the symmetry of loss (C-2L-2A). Dotted circles around the means represent statistically significant differences with pre-autotomy levels performance of that treatment. Major effects of time and treatment are based on the GLMMs tests (Table 1) and the dotted circles reflect post hoc tests (see Supplementary Table S3). Graph shows all gaits pooled. Plot shows data up to 24 h after autotomy . Sample size (N) is included on top of each treatment’s diagram. Figure was created in Microsoft Excel version 16.39 and Microsoft Power Point version 16.39 (URL: https://www.microsoft.com/en-us/microsoft-365/microsoft-office).

The type of leg lost did not affect locomotor performance after autotomy. Individuals that lost two locomotor or two sensory legs did not experience declines in either velocity or acceleration after autotomy. Further, there were no significant treatment × time interactions (*P* > 0.05 for both comparisons) (Fig. 3, Table 1, Supplementary Table S1).

#### Asymmetry of loss

**Figure 4:**
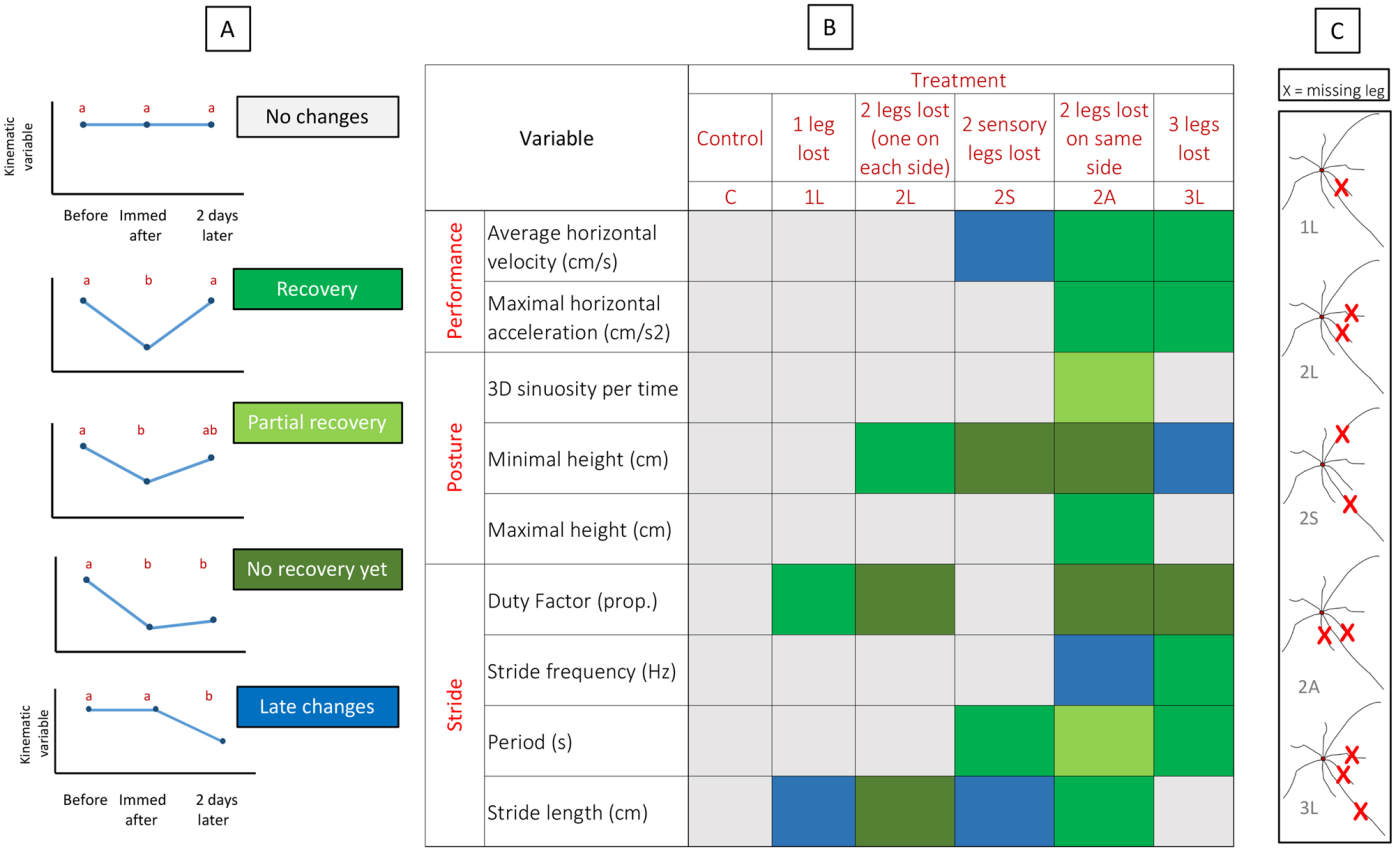
Summary of kinematic changes after leg loss in *Prionostemma* sp.1 harvestmen. (**A**) Possible patterns of kinematic changes. Different colors correspond to different patterns. (**B**) Post hoc Tukey results of the GLMs showing within treatment comparisons. Color codes corresponding to (**A**) reflect statistical analysis (see Supplementary Table S3). (**C**) schematic representation of the experimental treatments. X = leg loss. Figure was created in Microsoft Excel version 16.39 and Microsoft Power Point version 16.39 (URL: https://www.microsoft.com/en-us/microsoft-365/microsoft-office).

Immediately after autotomy, individuals that lost two locomotor legs on the same side (2A treatment, Fig. 1) experienced decreases in mean velocity and acceleration of 43% and 46%, respectively (Fig. 3, GLMM post hoc tests: *P* < 0.05). When comparing Control, 2L and 2A treatments we found significant treatment × time interactions for both velocity and acceleration (*P* = 0.02 and *P* = 0.04, respectively, Table 1, Supplementary Table S2). Despite these decreases, 2A individuals recovered performance to pre-autotomy levels after two hours (Figs. 3, 4, Supplementary Video S1, post hoc tests: *P* > 0.05).

### Stride and postural kinematics

#### Number of legs lost

The number of legs lost affected stride length and frequency. Immediately after autotomy, harvestmen that lost three legs moved with fewer strides (i.e. with a reduced stride frequency). However, stride frequency increased back to pre-autotomy levels after 2 days for 3L harvestmen (Fig. 4, Supplementary Tables S1, S3). Stride period and duty factor showed the opposite pattern: increased immediately after autotomy, but decreased to pre-autotomy levels over time (Fig. 4). Harvestmen that lost one or two locomotor legs (1L and 2L, respectively) also increased their duty factor (Fig. 4). Additionally, 2L and 3L harvestmen moved with a lower minimal height (Fig. 4), suggesting a more crouched posture after autotomy. Contrary to 3L harvestmen, 1L and 2L individuals had longer strides length after autotomy (Fig. 4). Together, these patterns suggest that modifications in stride and postural kinematics are dependent on the intensity of autotomy, and become more acute with the number of legs lost.

#### Type of missing leg

Harvestmen that lost two sensory legs (2S) displayed changes in stride period, stride length, and minimal height after autotomy (Fig. 4, Supplementary Table S1). Thus, after leg loss, 2S individuals moved with slower and longer strides, as well as with a more crouched posture. These changes resemble those of 2L animals (see above), with the exception of the changes in stride period, which recovered to pre-autotomy levels 2 days later.

#### Asymmetry of loss

Animals that lost two locomotor legs on the same side (2A) displayed changes in all stride and postural variables examined (Fig. 4, Supplementary Table S1). These harvestmen started walking with longer, slower and fewer strides, as well as a more crouched posture with a more sinuous trajectory, compared with their pre-autotomy movement patterns. Over time, only some of these variables went back to pre-autotomy levels (Fig. 4). However, no clear pattern of recovery occurred for stride and postural kinematics for 2A individuals.

### Gait types

Locomotor performance consistently differed between gait types. Running and stotting had higher velocity and acceleration than bobbing and walking. These differences occurred both before and after autotomy, as well as in every time frame analyzed (see Supplementary Fig. S2 and Table S3). The six treatments analyzed showed the same pattern of consistent differences between fast and slow gaits over time, as there was no significant interaction regarding gait pair in any model (Table 1).

**Figure 5:**
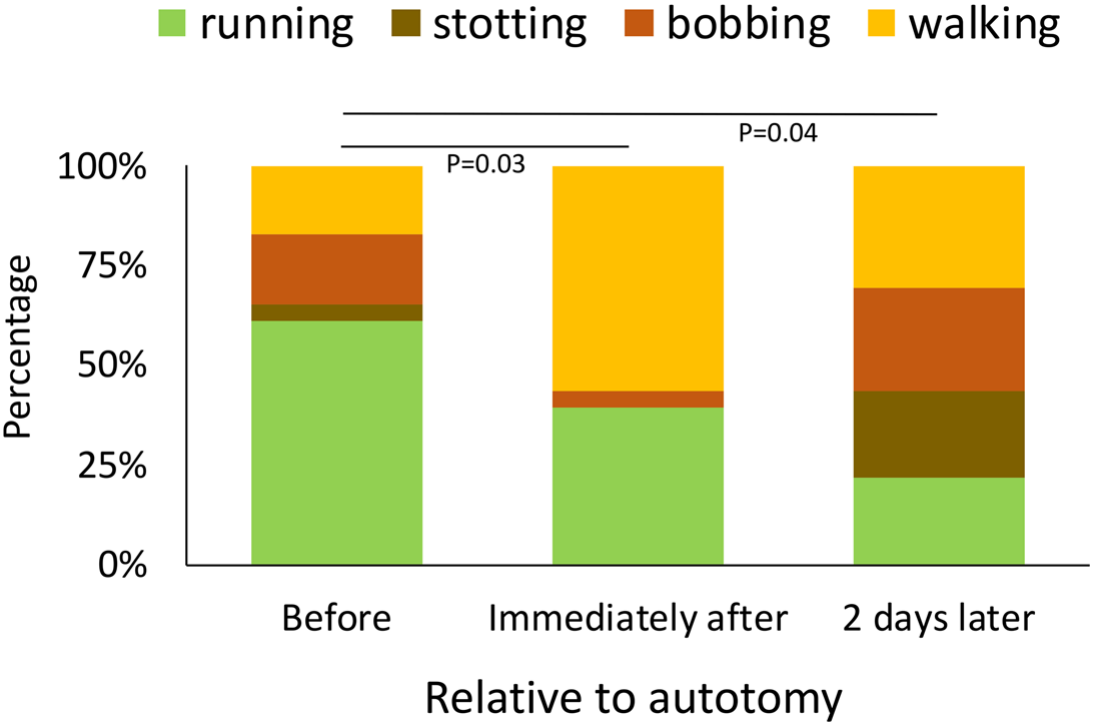
Frequency of gait types performed over time by *Prionostemma* sp.1 harvestmen in individuals that lost three legs (3L treatment) (n = 23). See “Methods” and Escalante et al.^35^ for further description of the types of gaits. *P* values are from independence chi-squares (Table 2). Similar patterns were found for 2S and 2A treatments (see “Results” and Supplementary Table S3). Figure was created in Microsoft Excel version 16.39 (URL: https://www.microsoft.com/en-us/microsoft-365/microsoft-office).

Across trials, running was the most commonly performed gait, whereas stotting was the least common (Table 2). The frequency of individuals performing a given gait type did not vary after autotomy for Control, 1L, or 2L animals. However, in the 2A and 3L treatments, the number of individuals walking increased after autotomy, and decreased for running (Fig. 5, Table 2). Gait frequency did not return to pre-autotomy levels (Fig. 5, Table 2). On the other hand, harvestmen that lost two sensory legs showed a different pattern, in which the proportion of individuals bobbing increased over time and did not decrease to pre-autotomy levels (Table 2).

**Table 2:** Gait types performed over time by *Prionostemma* sp.1 harvestmen.

Treatment/gait	Time relative to autotomy							
	Before	Immediately after	Immediate changes (P)	2 h later	6 h later	24 h later	2 days	Changes over time (P)
**C—Control (n = 24)**								
Running	13	14	0.82	14	16	11	10	0.33
Stotting	4	2		1		2	3	
Bobbing	2	3		5	2	1	7	
Walking	5	5		4	6	7	4	
**1L—1 leg lost (n = 21)**								
Running	8	13	0.4	13	14	5	5	0.77
Stotting	3	1		4	2	3	3	
Bobbing	4	2		2	2	3	1	
Walking	6	5		2	3	6	3	
**2L—2 locomotor legs lost (n = 23)**								
Running	13	7	0.23	8	7	9	11	0.14
Stotting	1	4		4	5	1	5	
Bobbing	4	4		2		1		
Walking	5	8		9	11	12	7	
**2S—2 sensory legs lost (n = 23)**								
Running	16	11	0.09	13	15	5	4	**0.001**
Stotting	2					5	2	
Bobbing	1	4		4	1	7	12	
Walking	3	7		5	6	5	4	
**2A—2 locomotor legs lost on same side of the body (n = 22)**								
Running	19	8	**0.002**	11	9	9	9	**0.02**
Stotting				3	2	4	1	
Bobbing	1	1		1		3	4	
Walking	2	13		7	11	6	8	
**3L—3 legs lost (n = 23)**								
Running	14	9	**0.03**	6	8	6	5	**0.04**
Stotting	1			1	2		5	
Bobbing	4	1		4	2	4	6	
Walking	4	13		12	11	13	7	

Sample size of each treatment in shown in parenthesis.

The *P* values in the “Immediate changes” column were extracted from chi squares comparing gait types before and immediately after autotomy. *P* values in “Changes over time” are derived from chi-square analyses comparing between the number of individuals performing each gait before and 2 days after autotomy. Statistical significance at the at the *P* < 0.05 level is marked in bold.

### Changes in leg use

**Figure 6:**
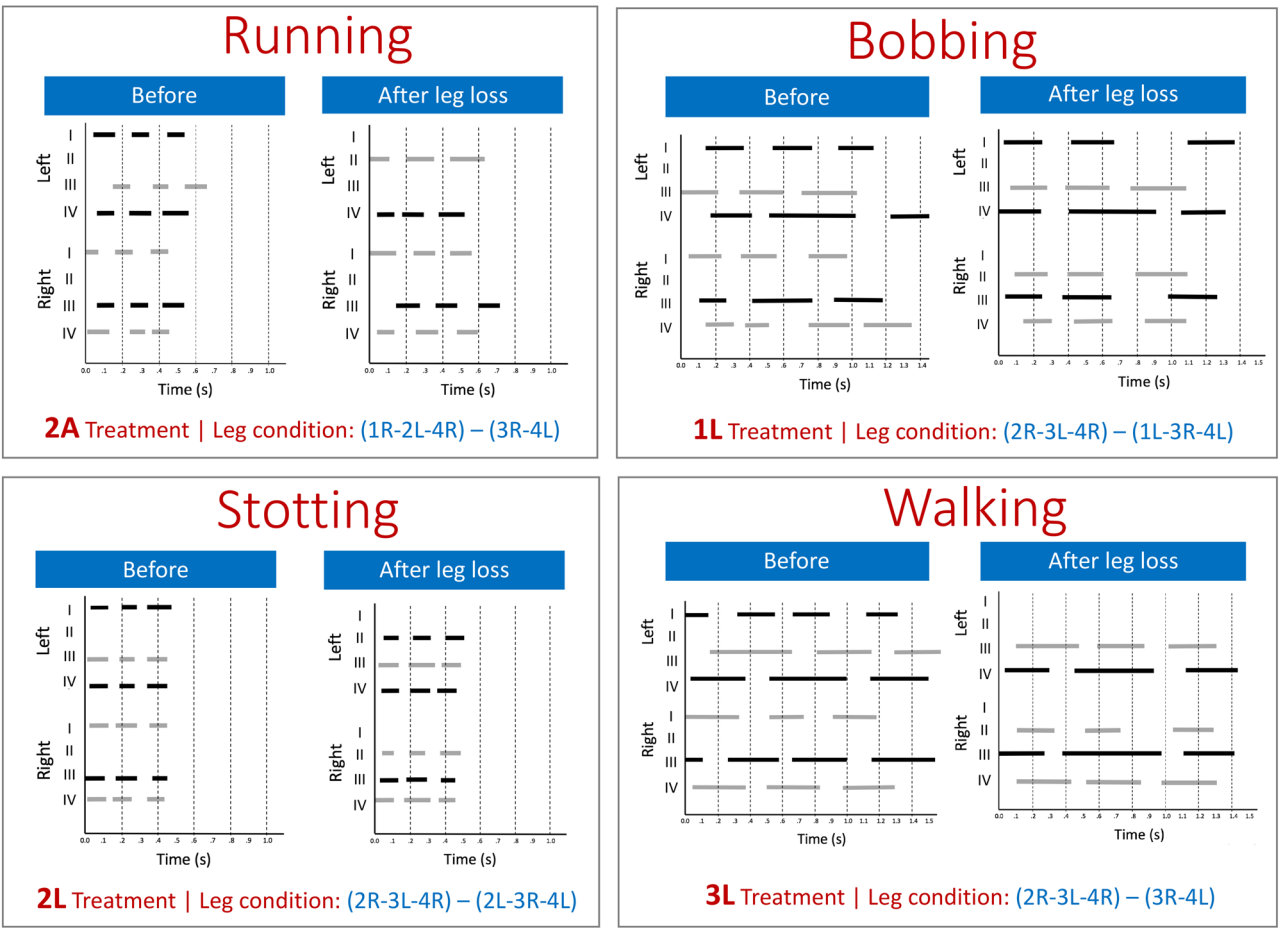
Gait diagrams of the four gaits performed by *Prionostemma* sp.1 harvestmen. Bars represent when each leg (1, 2, 3 or 4; from either the left or right side of the body) was on the ground. Each panel represents the same individual before and immediately after autotomy. These are representative harvestmen from different treatments that performed the same type of gait in both time frames. Note the changes in using legs II. Treatment nomenclature: the number represents the number of legs animals lost and the letter represents the type of loss (L: missing locomotor legs, S: missing sensory legs, and A: missing two locomotor legs in the same side of the body). Leg groups in parenthesis represent the two groups of leg tripods used during locomotion after leg loss. Black and grey color coding represent the original tripods when animals have an intact leg condition (Sensenig and Shultz^36^; Escalante et al.^35^). Legs II are not shown in the left side panels because harvestmen do not use them to move before autotomy in any treatment or gait. Control or 2S individuals are not shown as they never used legs II to move. Figure was created in Microsoft Power Point version 16.39 (URL: https://www.microsoft.com/en-us/microsoft-365/microsoft-office).

Autotomy affected which legs were used during locomotion (Fig. 6). Immediately after autotomy, leg II was used to move, which did not occur in intact or control individuals, which alternated between the 1R-3L-4R and 1L-3R-4L tripods (Fig. 6). Recruitment of leg II for locomotion occurred across all gait types (Fig. 6). After autotomy, 1L individuals started using the leg II (sensory leg) located next to the leg that was lost, matching the original tripod-gait (Fig. 6). Additionally, 2L individuals (missing both first legs) used one or both legs II during locomotion. The resulting alternating tripod gait for 2L individuals was 2L-3R-4L and 2R-3L-4R (Fig. 6). Harvestmen with an asymmetric loss (2A) used the leg II of the autotomized side to move. In that case, leg II replaced the lost leg III, instead of replacing leg I as in the 1L and 2L treatments. The new leg pattern for 2A individuals was 1R-2L-4R and 3R-4L (Fig. 6). Finally, 3L individuals showed major changes in their tripod gait after autotomy. They started using the remaining leg II to move, which replaced the missing leg I on the same side. Their new gait pattern was 3R-4L and 2R-3L-4R. Control and 2S animals did not display changes in the legs used to move over time.

## Discussion

### Consequences of leg loss and locomotor recovery

Overall, our findings demonstrate that harvestmen are robust to perturbations imposed by bodily damage, specifically leg loss. We found negative effects of autotomy on locomotor performance (velocity and acceleration) only in individuals that lost three legs total or two locomotor legs on the same side of the body. In contrast, performance was not affected by the loss of one or two locomotor legs or two sensory legs. Therefore, we found an effect of autotomy based on the number of legs lost and the asymmetry of autotomy, but not on the type of leg lost. It is possible that the immediate decrease in locomotor performance was due to balance disruptions of the standard alternating tripod gait used by harvestmen^35,36^, only after surpassing a certain threshold of damage. Autotomy can impede the ability for the remaining legs to alternate between stance and aerial phase, potentially by decreasing the torque produced by the power stroke from the body side with missing legs^18^. Ultimately, these changes will prevent animals from quickly moving away from potentially agonistic stimuli.

For situations in which performance decreased immediately after autotomy, recovery of initial levels of velocity and acceleration occurred within two days of leg loss. Overall, our findings indicate that harvestmen are capable of compensating for the permanent loss of legs. Additionally, our findings represent the quickest recovery of locomotor performance recorded in animals to date^7^.

Despite the observed rapid recovery of locomotor performance after autotomy, differences in the magnitude of leg loss may affect the survival of *Prionostemma* harvestmen. For instance, missing a large number of legs, or missing many legs on one side of the body only could impact locomotion severely, as found here. Individuals missing three or more legs or more than two legs on the same side were rare in our study population as well as in other species (I. Escalante, *unpublished*), suggesting that such levels of autotomy negatively affect survival. Consequently, the 2 days immediately after autotomy—i.e., before recovery—could be a particularly vulnerable period for these arachnids. The specific body location where loss happens might also influence the effects of autotomy on locomotion. Losing rear legs can modify gaits more substantially since legs IV showed a higher contact during stance phase when compared to hind legs. Our design did not test for this possibility. However, future research will address this topic.

### Compensation for leg loss

#### Kinematics of locomotion

Our findings suggest that recovery of locomotor performance is associated with fine-scale adjustments in stride and postural kinematics. Individuals that experienced extensive leg loss (i.e. 2A and 3L) reorganized their kinematics through subtle adjustments. These harvestmen increased stride frequency and decreased stride period and duty factor. In particular, duty factor shifts after leg loss occurred in all treatments except in individuals that lost the two sensory legs (2S), suggesting its importance for compensation. Changes in duty factor have important implications to the interchange of potential and kinetic energy (as well as elastic energy), the ground reaction force exerted on the legs^50^, and the energetics of locomotion, as^51^ found an increase in oxygen consumption after leg loss in crabs.

Additionally, changes in gait type, as well as the legs used for locomotion played a role in recovery. Similar mechanisms for compensation following autotomy have been reported for green anoles^16^ and leopard geckos^17,26^. Changes in the position and time of leg stance phases after autotomy in wolf spiders allowed them to avoid negative consequences of autotomy on running stability and performance^29^.

Given the absence of regeneration, harvestmen are expected to quickly adapt to leg loss. For species that regenerate appendages, their relative robustness to damage and the ability to recover has been found to correlate with the time needed to fully regenerate. These patterns have been found after tail regeneration in skinks^27^, lizards^28^, and geckos^26^. Our findings suggest that, in the absence of limb regeneration, kinematic and behavioral compensatory mechanisms might assist the recovery and improve robustness to bodily damage, as found for ants during the homing navigation after leg loss^25^.

#### Gait type

We found that the gait-specific variation in performance^35^ did not affect recovery. After autotomy, however, harvestmen did change the frequency of gait types used to move. Particularly, walking became more frequent after autotomy in animals that lost more than one leg. Additionally, bobbing became more common only in animals that lost both sensory legs. These changes in gait types used may reflect biomechanical and/or physiological constraints imposed by a new leg combination. Running gaits are strongly associated with elastic energy recovery^36^. Consequently, leg loss might affect the functionality and use of the spring-mass dynamics, as the symmetry of that recovery is disrupted. Regardless of this, individuals that experienced high levels of leg loss were less able to move using the conventional alternating tripod gait for harvestmen^36^, which may increase the frequency of non-running gaits. Additionally, losing legs decreased the number of spring-mass monopods acting on the total stiffness^50^ of the system. This makes more dynamic gaits (i.e. running and stotting) less favorable in terms of energetics and ground reaction forces^50^. Therefore, decreases in the stiffness of the overall locomotor system of harvestmen may explain why gaits such as bobbing and walking became more common after autotomy.

Alternatively, changing the gait type performed may be adaptive. The different gaits of *Prionostemma* harvestmen have been suggested to confer distinct benefits in terms of predator avoidance^35^ or represent different optima in the physiological costs of locomotion. Consequently, post-autotomy modifications in gait may reflect adaptive adjustments to changes in predation risk or costs of moving. Post-autotomy changes in anti-predatory behavior have also been interpreted as compensation in snails^52^, dragonfly larvae^53^, and lizards^16,54^. Whether changes in the gait type use are due solely to mechanical constraints or if bodily damage promotes behavioral plasticity is unclear and can be addressed in future research.

#### Legs used for locomotion

Plasticity in leg function can allow harvestmen to compensate for the effects of leg loss. The ‘sensory’ legs acquired a locomotor function in this study. In animals whose performance did not change post-autotomy (1L, 2L, and 2S), individuals began using the sensory legs in locomotion immediately after leg loss, which does not occur in intact eight-legged animals^35^. These findings are novel in arthropods and suggest a redundant locomotory function to the sensory legs. Sensory legs thus not only serve an “antenniform” function^55^ but also serve as backup locomotor legs in case of leg loss. Having this redundant function is only possible since the ‘sensory appendages’ of harvestmen retain the same leg shape, as opposed to differently-shaped appendages in insects or the “antenniform” legs of whip spiders. Hence, maintaining that shape over evolutionary time will benefit the robustness for leg loss in these arthropods. Previous research has also reported the recruitment of food-handling appendages for locomotion after leg loss, including the pedipalps in tarantulas^21^ and the chela in crabs^24^.

The specific kinematic mechanisms promoting recovery remain unknown for these harvestmen. Changes in the use of the recently-recruited leg II for locomotion could contribute to the locomotor recovery observed here. Additionally, after leg loss harvestmen could modify the stance phase of that leg, as well as of other legs. Along with changes in minimal height during locomotion, we found changes in duty factor over time for one leg. Future studies can address the quantitative variation across treatments, time, and gaits of postural and stride kinematics of all legs. This could elucidate the fine-scale mechanisms allowing locomotor recovery. This will require higher resolution information about what each leg is doing over time. However, since *Prionostemma* harvestmen legs are so thin we were not able to automatically track their movement.

Leg multi-functionality raises the possibility of movement and sensory trade-offs after autotomy, suggesting that compensation may have other consequences. In this experiment, harvestmen that lost both sensory legs were the only group that increased the proportion of bobbing, even though they did not show other changes in performance. This suggests that bobbing can trade-off with sensory performance, and 2S harvestmen are relying on this gait more often potentially to gather information from the environment. However, we do not have evidence for this claim, but future research can address the interactive effects of autotomy on locomotion and foraging, sensory abilities, survival, or reproduction.

## Conclusions

Our findings suggest that harvestmen are robust to bodily damage. For most conditions of leg loss tested here, harvestmen rapidly altered features of their locomotion to maintain and/or regain performance. In extreme cases (asymmetric leg loss, loss of three legs), locomotor performance decreased after leg loss, but harvestmen were still able to recover relatively quickly. We found that adjustments in the kinematics of locomotion (including changes in duty factor, stride frequency, stride period, and body height) and changes in the legs used to move were correlated with recovery of locomotor performance. Bodily damage due to autotomy is widespread across taxa and we suggest that many species may have evolved mechanisms to compensate for the diminished performance caused by injuries. Despite the fact that our analyses did not allow us to unravel the kinematic mechanisms promoting recovery, we suggest that compensation is important, yet often overlooked facet of fitness. Accordingly, comparative studies of animals with different body plans, natural histories and physiologies are important to understanding the evolution of responses to limb loss and other forms of bodily damage.

## Supplementary information

Supplementary Video 1.

Supplementary Information.

## Data Availability

The complete dataset included in this study is available on Dryad here.
