# Supplementary material for: Rapid recovery of locomotor performance after leg loss in harvestmen
Source: Sci Rep. 2020 Aug 13;10:13747. doi: 10.1038/s41598-020-70557-2 (PMC7426809; doi:10.1038/s41598-020-70557-2)
Supplement: Supplementary file 2 — Supplementary Information. [file 41598_2020_70557_MOESM2_ESM.pdf]

Supplementary information

Manuscript tittle:

Rapid recovery of locomotor performance after leg loss in harvestmen

Authors:

Ignacio Escalante[1,*], Marc A Badger[2,3], and Damian O Elias[1]

[1]Department of Environmental Sciences, Policy, & Management. University of California - Berkeley, California, USA. 94720.

[2] Department of Integrative Biology, University of California - Berkeley, California, USA. 94720.

[3] current address: Department of Computer and Information Science, University of Pennsylvania - Philadelphia, Pennsylvania, USA. 19104.

[*] Corresponding author; iescalante@berkeley.edu. 130 Mulford Hall. Berkeley, CA, USA. 94720.

Supplementary Video S1

Sample movement of *Prionostemma* sp. 1 harvestmen before and after leg loss (autotomy). Two different animals that experienced different levels of autotomy are shown. Each individual is shown before, immediately after autotomy and 2 days later. See Methods for further description of the treatments. The video is available here.

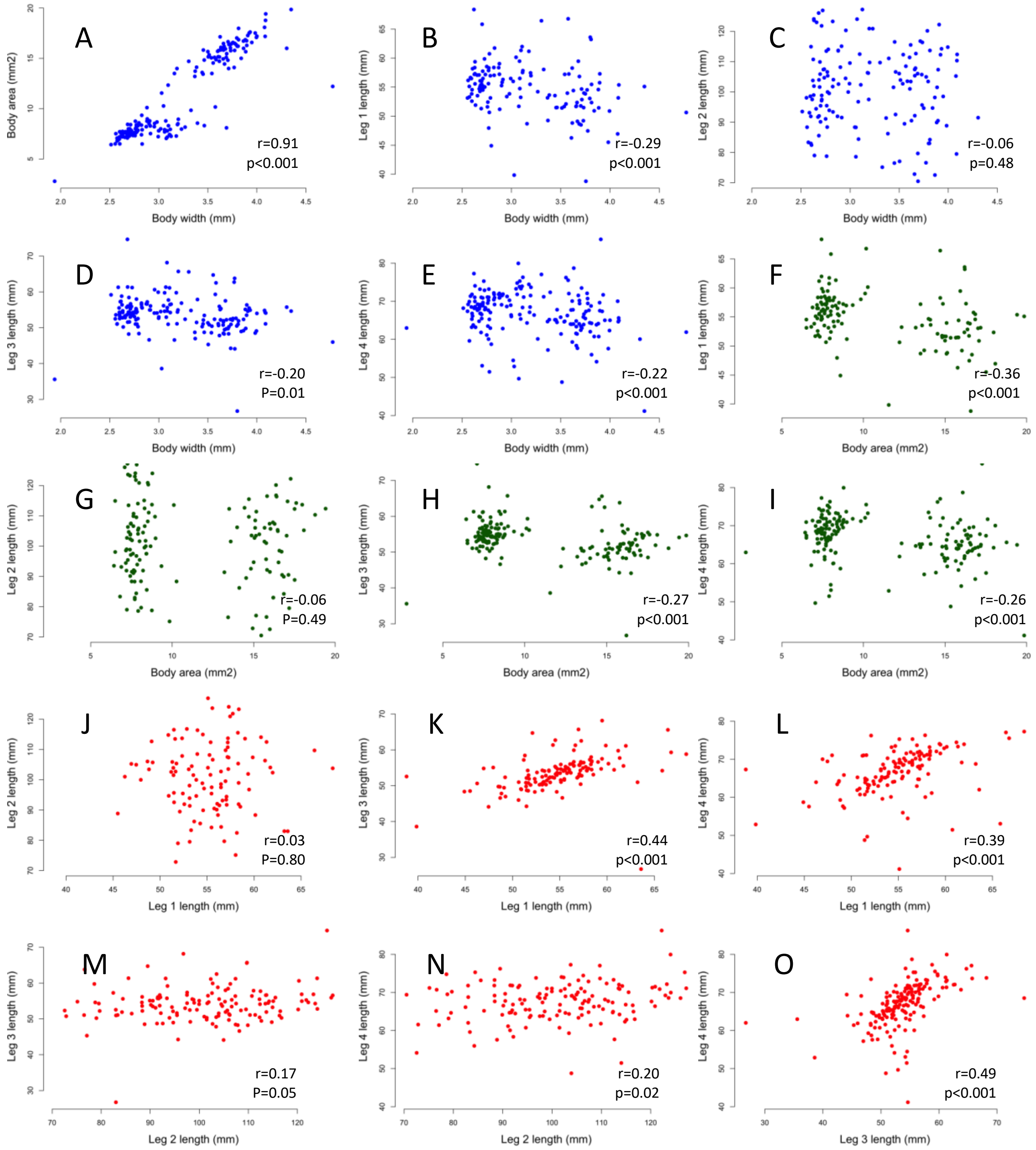

Supplementary figure 1. Morphological features of *Prionostemma* sp.1 harvestmen. Correlation coefficients (r) and associated *P* are shown. Clustering, particularly in panels A, and F through I represent variation based on sex, with females being larger than males. Figure was created in R (R Development Core Team 2018, URL: https://www.r-project.org/).

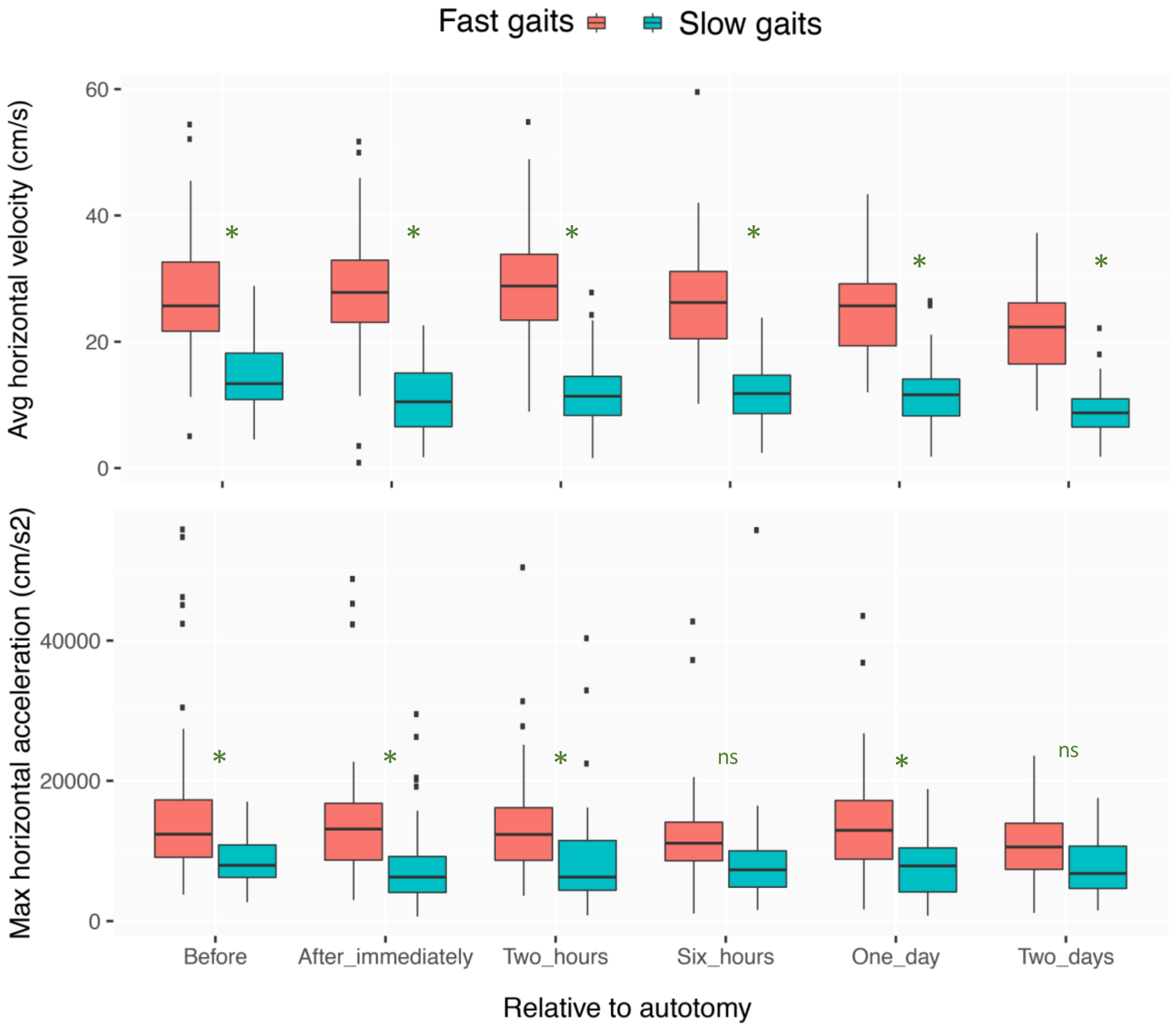

Supplementary figure 2. Velocity and acceleration as measures of locomotor performance in *Prionostemma* sp.1 harvestmen at different times, grouped fast gaits (running and stotting), and slow gaits (bobbing and walking). For detailed descriptions of the gaits see Methods and (Escalante et al. 2019). Boxplots center lines represent the median ± 25% quartiles. Green asterisks denote statistical differences between fast and slow gaits at a given time. “ns” = no differences between groups. All comparisons at the $P = 0.05$ level. Velocity was similar between running (mean ± sd= 28.14 ± 8.59 cm/s, range= 12.81-54.40, n=107) and stotting (28.00 ± 8.07 cm/s, range= 7.59-38.78, n=12) across all time points, and between bobbing (13.62 ± 4.26 cm/s, range= 4.56-19.64, n=24) and walking (12.67 ± 3.38 cm/s, range= 4.53-19.58, n=34). Figure was created in R (R Development Core Team 2018, URL: https://www.r-project.org/).

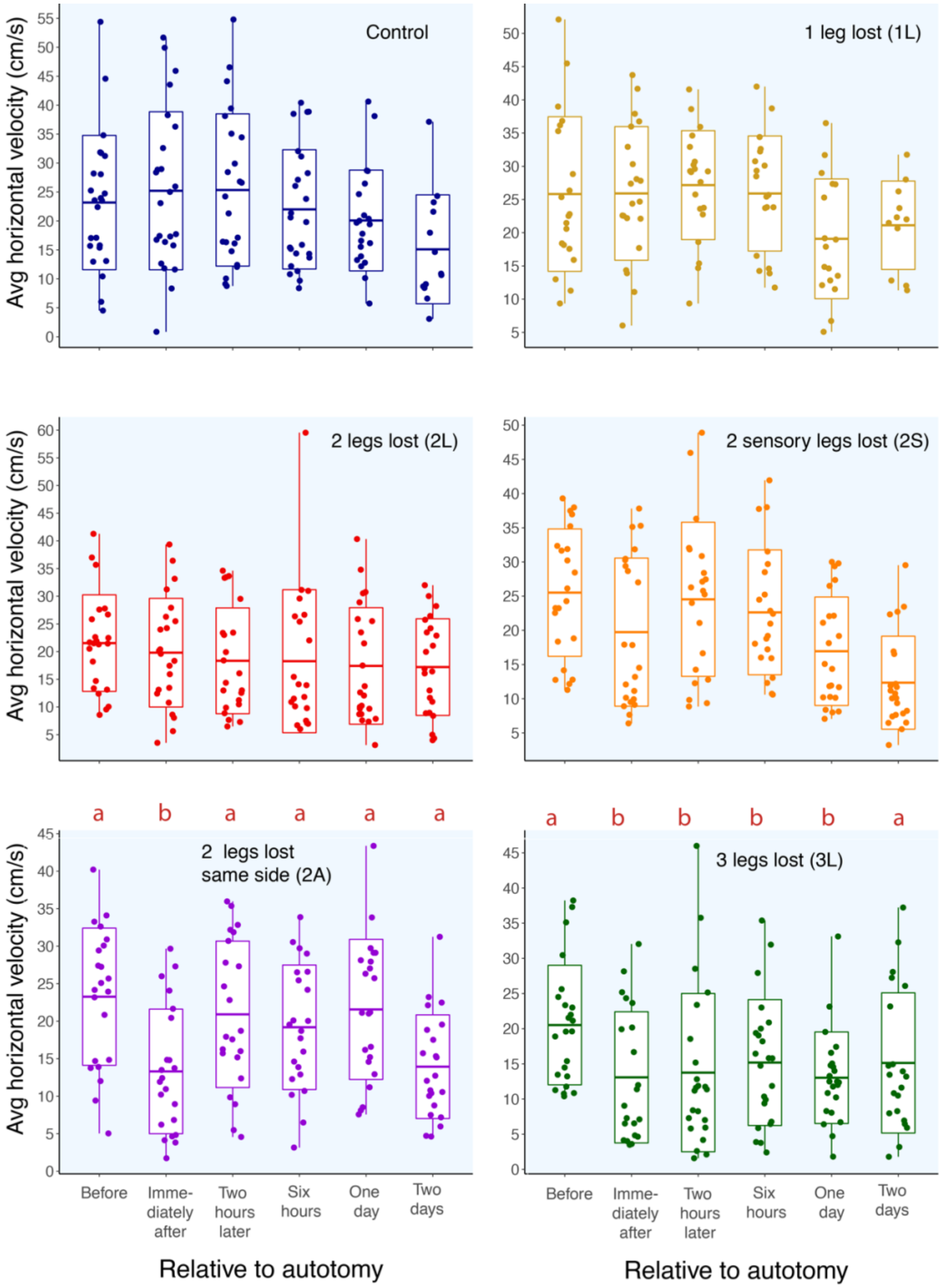

Supplementary figure 3. Jitter plots of the average horizontal velocity (cm/s) of *Prionostema* harvestmen over time. Boxplot center lines represent the mean ± 1 standard deviation. Each panel show a different treatment based on the leg condition after experimentally induced autotomy. See Methods for further description of the treatments and Table S1 for sample sizes. Statistical differences based on GLMMs were found only in the bottom two panels, where letters denote differences. See Table 1 for statistical results. Figure was created in R (R Development Core Team 2018, URL: https://www.r-project.org/).

Supplementary table 1

Summary of the performance, postural and stride kinematic variables of the locomotion of *Prionostemma* sp.1 harvestmen. The table reports sample size (n), mean, standard deviation (sd), and range for all treatments across time. Gait pair pools fast (running and stotting) and slow (bobbing and walking) gaits. See Methods and Escalante et al. (2019) for further descriptions of the gaits.

Variable	Gait pair	Time	Control				1 leg lost (1L)				2 legs lost (one on each side) (2L)				2 sensory legs lost (2S)				2 legs lost (on the same side) (2A)				3 legs lost (3L)			
			n	mean	sd	range	n	mean	sd	range	n	mean	sd	range	n	mean	sd	range	n	mean	sd	range	n	mean	sd	range
PERFORMANCE VARIABLES																										
Average horizontal velocity (cm/s)	fast	Before	17	27.9	10.2	41.4	10	32.8	11.7	36.2	14	25.7	8.0	28.1	18	28.3	7.9	28.0	19	24.9	8.7	35.2	15	24.3	7.9	25.0
		After	16	30.5	13.8	50.8	14	30.6	8.1	29.8	11	27.4	7.4	25.9	11	28.9	7.1	23.3	8	22.2	5.8	16.2	9	20.6	8.8	28.5
		After 2hrs	14	32.3	11.8	40.0	16	30.3	5.4	18.8	12	25.1	7.6	22.1	13	31.6	7.8	24.9	14	24.9	8.7	27.0	7	25.8	12.2	34.8
		6 hrs	16	25.8	10.2	29.1	14	30.1	5.6	18.3	10	27.8	13.0	47.7	15	26.3	8.1	26.0	11	25.0	6.6	23.7	10	23.2	6.5	19.6
		1 day	13	23.1	8.7	27.4	8	26.3	6.9	21.9	10	27.5	7.4	26.6	10	24.0	5.4	14.9	13	26.8	7.4	28.2	6	20.3	7.3	21.1
		2 days	6	21.7	9.6	28.0	8	24.5	3.8	11.1	15	21.8	6.2	21.0	6	21.9	4.8	13.0	10	18.7	6.0	20.7	10	22.8	8.9	25.6
	slow	Before	7	11.7	4.9	12.6	10	18.8	6.2	19.5	9	15.1	5.4	13.3	4	13.0	0.8	2.0	3	12.7	2.9	5.4	8	13.4	3.7	9.2
		After	8	14.6	3.3	9.4	7	16.6	6.4	16.6	12	12.9	5.7	16.9	11	10.5	3.1	11.5	14	8.2	4.2	13.1	12	7.5	4.6	16.3
		After 2hrs	9	14.5	5.5	15.5	4	14.5	3.8	9.3	10	10.2	2.7	7.9	8	13.1	4.2	12.2	8	13.9	7.6	23.2	16	8.5	5.4	21.8
		6 hrs	8	14.4	5.3	15.4	5	14.2	1.7	4.8	11	9.6	3.1	9.4	6	13.6	3.2	8.2	11	13.4	5.2	16.9	13	9.1	4.7	14.0
		1 day	8	15.1	6.5	20.7	9	12.6	4.6	14.1	13	9.6	3.3	14.4	12	11.1	3.8	14.1	9	14.0	6.2	18.2	17	10.5	3.9	13.2
		2 days	7	9.5	4.6	14.9	3	12.1	0.7	1.5	7	7.3	2.9	7.7	16	8.8	2.6	8.9	12	10.0	5.0	17.5	11	8.2	4.0	12.9
Maximal horizontal acceleration (cm/s2)	fast	Before	15	199	134.6	481	9	190	140.4	470	14	129	53.4	198	18	123	46.5	164	19	122	65.4	267	15	165	125.7	393
		After	13	186	136.3	458	11	138	47.6	127	11	145	107.5	392	11	139	27.2	85	6	92	42.2	120	9	96	54.7	138
		After 2hrs	13	140	57.0	195	15	149	70.0	267	11	106	56.8	186	13	117	33.2	110	13	160	124.5	468	6	114	39.3	108
		6 hrs	14	130	79.4	303	14	113	44.0	147	8	103	49.7	140	14	124	27.3	100	11	118	48.4	165	9	139	113.6	372
		1 day	12	136	96.7	352	8	170	112.9	346	9	148	59.8	191	9	123	56.9	165	12	109	40.6	116	5	157	49.9	125
		2 days	6	121	33.3	92	8	126	55.6	158	14	99	45.7	134	5	155	48.3	118	10	79	37.8	132	8	104	50.2	125
	slow	Before	7	81	29.6	91	8	84	24.7	75	9	81	40.8	131	4	76	38.4	68	3	80	16.5	33	6	96	56.9	128
		After	7	87	56.4	170	7	122	86.8	265	12	80	50.3	176	11	92	76.6	238	13	58	27.2	91	12	51	25.2	82
		After 2hrs	9	75	34.1	105	4	71	32.2	74	10	111	109.3	372	8	99	61.1	189	8	92	102.9	303	15	74	48.2	154
		6 hrs	7	87	42.8	133	5	87	41.8	99	11	62	32.9	96	6	66	20.8	59	9	83	42.7	118	12	105	145.6	534

		1 day	7	108	43.6	123	9	90	53.6	163	13	69	39.3	115	12	58	30.2	106	9	73	33.7	91	15	91	44.1	173
		2 days	6	76	36.8	86	3	93	49.2	93	7	49	10.5	27	16	83	40.3	137	12	77	46.0	160	9	66	27.4	97
POSTURE VARIAVBLES																										
3D sinuosity per time	fast	Before	15	1.5	0.6	2.2	9	1.8	0.5	1.9	14	1.4	0.5	1.9	18	1.4	0.4	1.3	19	1.2	0.4	1.3	15	1.2	0.3	1.1
		After	13	1.4	0.5	1.7	13	1.5	0.5	1.7	10	1.4	0.5	1.6	11	1.5	0.3	1.1	8	1.2	0.6	2.0	9	1.0	0.4	1.6
		After 2hrs	14	1.7	0.7	2.1	15	1.6	0.4	1.2	12	1.5	0.4	1.4	13	1.5	0.3	1.1	13	1.4	0.6	2.3	7	1.3	0.5	1.7
		6 hrs	16	1.6	0.7	2.3	14	1.5	0.3	0.9	8	1.5	0.5	1.2	15	1.4	0.4	1.5	11	1.4	0.4	1.6	10	1.5	0.4	1.2
		1 day	12	1.2	0.5	1.9	8	1.4	0.3	0.7	10	1.5	0.4	1.3	9	1.3	0.3	0.8	13	1.6	0.5	1.2	6	1.4	0.5	1.5
		2 days	6	1.1	0.5	1.2	8	1.6	0.6	1.8	15	1.5	0.4	1.4	5	1.1	0.3	0.6	10	1.2	0.4	1.4	9	1.7	0.6	1.9
	slow	Before	7	0.6	0.2	0.7	9	1.1	0.6	1.8	9	0.9	0.3	1.0	4	0.7	0.2	0.5	3	0.6	0.2	0.4	8	0.9	0.4	0.9
		After	8	1.1	0.5	1.2	7	0.9	0.4	0.9	12	0.8	0.3	1.0	11	0.6	0.2	0.8	14	0.5	0.2	0.5	12	0.7	0.7	2.6
		After 2hrs	9	0.9	0.3	0.8	4	0.8	0.3	0.7	10	0.8	0.3	0.8	8	0.8	0.5	1.4	8	0.8	0.4	1.2	16	0.7	0.5	1.9
		6 hrs	8	1.0	0.7	2.0	5	0.9	0.2	0.4	11	0.6	0.2	0.8	6	0.8	0.3	0.6	11	0.9	0.5	2.1	13	0.6	0.3	1.2
		1 day	7	0.8	0.5	1.1	9	0.7	0.3	0.8	13	0.7	0.3	1.1	12	0.7	0.3	0.9	9	0.9	0.3	0.9	17	0.7	0.3	0.8
		2 days	7	0.6	0.3	0.7	2	0.6	0.0	0.1	7	0.7	0.3	0.8	16	0.8	0.2	0.8	12	0.7	0.2	0.8	11	0.8	0.5	1.8
Minimal height (cm)	fast	Before	15	0.5	0.5	2.1	9	0.3	0.3	0.9	14	0.7	0.4	1.5	18	0.8	0.5	1.5	19	1.0	0.5	1.7	15	0.5	0.5	1.6
		After	16	0.5	0.6	1.8	13	0.4	0.2	0.7	11	0.3	0.3	1.1	11	0.2	0.2	0.8	7	0.3	0.5	1.4	9	0.1	0.3	1.1
		After 2hrs	14	0.8	0.3	1.1	15	0.3	0.3	1.4	11	0.2	0.2	0.7	13	0.6	0.6	1.8	14	0.1	0.4	1.8	7	0.2	0.3	0.9
		6 hrs	15	1.0	0.5	1.6	14	0.6	0.3	1.2	10	0.2	0.4	1.2	15	0.6	0.5	1.6	11	0.1	0.4	1.6	9	0.1	0.2	0.7
		1 day	12	0.4	0.4	1.5	8	0.2	0.3	1.0	10	0.3	0.3	1.1	10	0.1	0.1	0.3	13	0.0	0.2	0.8	6	0.0	0.1	0.3
		2 days	6	0.6	0.3	0.7	7	0.1	0.2	0.7	14	0.3	0.3	1.0	5	0.5	0.5	1.3	10	0.2	0.3	0.9	9	0.2	0.2	0.7
	slow	Before	7	1.0	0.4	1.1	9	0.9	0.4	1.4	9	0.9	0.6	1.7	4	0.9	0.4	0.9	3	0.9	0.8	1.7	7	0.3	0.4	1.0
		After	7	0.8	0.5	1.3	7	0.7	0.5	1.1	12	0.5	0.5	1.5	11	0.5	0.4	1.3	14	0.4	0.4	1.4	12	0.4	0.4	1.3
		After 2hrs	8	0.7	0.4	1.1	4	0.4	0.5	1.1	10	0.7	0.7	2.1	8	0.6	0.7	1.7	8	0.4	0.4	1.0	16	0.2	0.3	1.1
		6 hrs	7	0.8	0.3	1.0	5	0.4	0.2	0.5	11	0.5	0.5	1.4	6	0.5	0.5	1.1	10	0.5	0.4	1.5	12	0.3	0.4	1.5
		1 day	7	0.6	0.4	1.2	9	0.4	0.4	1.5	12	0.7	0.6	1.8	12	0.5	0.5	1.5	9	0.2	0.4	1.2	17	0.1	0.1	0.5
		2 days	6	0.8	0.3	0.7	3	1.2	0.5	1.0	7	0.4	0.5	1.4	16	0.2	0.2	0.7	12	0.6	0.4	0.9	11	0.2	0.3	1.1
Maximal height (cm)	fast	Before	17	3.0	0.7	3.1	10	3.0	0.7	2.0	14	2.8	0.2	0.8	18	2.6	0.4	1.5	19	2.7	0.3	1.3	15	2.7	0.3	1.2
		After	16	2.5	0.8	3.5	14	3.1	0.5	1.6	11	3.1	1.1	4.1	11	2.8	0.3	1.0	8	2.5	0.4	1.1	9	2.4	0.5	1.5
		After 2hrs	14	2.9	0.4	1.3	16	2.7	0.7	3.0	12	3.0	0.5	1.5	13	2.9	0.4	1.4	14	3.0	1.3	5.1	7	2.9	0.5	1.3
		6 hrs	16	2.9	0.4	1.4	14	3.1	0.4	1.3	10	3.2	0.9	3.2	15	2.7	0.4	1.4	11	2.5	0.3	1.1	10	2.8	0.6	2.1
		1 day	13	2.7	0.4	1.2	8	3.0	0.3	0.9	10	2.9	0.4	1.2	10	2.7	0.4	1.2	13	2.6	0.4	1.2	6	2.8	1.7	4.7
		2 days	6	2.7	0.4	0.9	8	2.4	0.7	2.1	15	2.9	0.5	1.9	6	2.6	0.4	1.1	10	2.4	0.6	1.8	10	2.4	0.4	1.5

	slow	Before	7	2.3	0.4	1.0	10	2.6	0.5	1.9	9	2.5	0.4	1.3	4	2.4	0.2	0.3	3	2.5	0.6	1.0	8	2.5	0.3	1.1
		After	8	2.7	0.7	2.0	7	2.5	0.4	1.1	12	2.6	0.4	1.4	11	2.4	0.5	1.7	14	2.0	0.2	0.9	12	2.2	0.5	1.6
		After 2hrs	9	2.6	0.5	1.5	4	2.3	0.3	0.6	10	2.6	0.3	0.9	8	2.6	0.5	1.4	8	2.5	0.5	1.6	16	2.1	0.7	2.7
		6 hrs	8	2.4	0.6	1.6	5	2.5	0.2	0.5	11	2.3	0.3	0.9	6	2.1	0.3	0.7	11	2.2	0.2	1.0	13	1.8	0.5	1.6
		1 day	8	2.2	0.5	1.3	9	2.5	0.5	1.6	13	2.4	0.5	2.0	12	2.3	0.4	1.3	9	2.2	0.4	1.2	17	2.1	0.7	3.0
		2 days	7	2.7	1.5	4.2	3	2.6	0.4	0.8	7	2.2	0.2	0.4	16	2.2	0.3	1.3	12	2.2	0.3	1.1	11	2.4	1.5	5.6
STRIDE VARIABLES																										
Duty Factor (prop.)	fast	Before	17	0.48	0.1	0.31	11	0.45	0.1	0.19	14	0.45	0.1	0.29	18	0.45	0.1	0.33	19	0.41	0.1	0.32	15	0.47	0.1	0.30
		After	16	0.47	0.1	0.28	14	0.51	0.1	0.23	11	0.58	0.1	0.25	11	0.43	0.1	0.36	8	0.49	0.1	0.29	9	0.59	0.1	0.46
		After 2hrs	15	0.45	0.1	0.27	17	0.54	0.1	0.28	12	0.57	0.1	0.31	13	0.37	0.1	0.30	14	0.50	0.1	0.48	7	0.53	0.1	0.22
		6 hrs	16	0.43	0.1	0.29	16	0.46	0.1	0.28	12	0.58	0.1	0.23	15	0.40	0.1	0.36	11	0.49	0.1	0.23	10	0.55	0.1	0.26
		1 day	13	0.46	0.1	0.43	8	0.41	0.1	0.23	10	0.56	0.1	0.19	10	0.42	0.1	0.25	13	0.51	0.1	0.30	6	0.59	0.1	0.27
		2 days	6	0.41	0.1	0.20	8	0.48	0.1	0.28	16	0.55	0.1	0.28	6	0.49	0.1	0.23	10	0.53	0.1	0.23	11	0.55	0.1	0.34
	slow	Before	7	0.56	0.1	0.30	10	0.48	0.0	0.15	9	0.54	0.1	0.36	4	0.57	0.1	0.21	3	0.61	0.0	0.08	8	0.58	0.1	0.15
		After	8	0.50	0.1	0.33	7	0.57	0.1	0.30	12	0.74	0.1	0.31	11	0.52	0.1	0.26	14	0.68	0.1	0.35	14	0.76	0.1	0.31
		After 2hrs	9	0.54	0.1	0.39	4	0.57	0.1	0.12	11	0.72	0.1	0.38	9	0.49	0.1	0.22	8	0.65	0.2	0.44	16	0.69	0.2	0.67
		6 hrs	8	0.53	0.1	0.33	5	0.60	0.1	0.19	11	0.73	0.1	0.32	7	0.57	0.1	0.16	11	0.62	0.1	0.29	13	0.67	0.1	0.41
		1 day	8	0.51	0.1	0.22	9	0.60	0.1	0.22	13	0.75	0.1	0.24	12	0.52	0.1	0.18	9	0.60	0.1	0.47	17	0.71	0.1	0.38
		2 days	8	0.59	0.1	0.38	4	0.52	0.2	0.38	7	0.74	0.1	0.21	16	0.51	0.1	0.32	12	0.60	0.1	0.23	12	0.71	0.1	0.24
Stride frequency (Hz)	fast	Before	17	4.86	1.6	5.52	11	4.91	0.9	3.34	14	4.23	1.2	3.73	18	4.70	1.3	3.91	19	4.25	1.2	5.29	15	3.98	1.0	3.05
		After	16	4.88	1.6	5.50	14	4.97	1.4	4.13	11	4.97	0.7	2.63	11	4.97	1.3	4.20	8	4.50	1.6	4.63	9	3.41	1.3	4.17
		After 2hrs	15	5.04	1.5	4.69	17	5.06	0.8	2.62	12	4.50	1.2	3.29	13	4.89	0.7	2.75	14	4.63	1.2	3.62	7	4.35	1.7	4.37
		6 hrs	16	4.62	1.6	5.83	16	5.41	0.9	3.47	12	5.06	1.2	3.65	15	4.21	1.0	3.19	11	4.94	1.1	3.65	10	4.32	1.1	2.94
		1 day	13	4.17	1.5	4.63	8	5.30	1.0	2.65	10	5.05	1.0	3.24	10	4.43	1.1	3.26	13	5.38	1.8	7.32	6	3.90	1.3	3.75
		2 days	6	3.89	0.7	2.03	8	5.16	1.0	2.68	16	4.95	1.1	3.62	6	4.30	1.0	2.69	10	4.57	1.3	3.52	11	4.57	1.2	3.72
	slow	Before	7	2.43	0.8	2.18	10	3.77	1.5	4.14	9	2.87	0.8	2.43	4	2.54	0.2	0.56	3	3.33	1.6	3.26	8	2.59	0.7	2.13
		After	8	2.81	1.0	3.45	7	3.34	1.1	2.53	12	2.65	1.2	3.72	11	2.20	0.8	3.14	14	2.33	1.0	3.68	14	1.73	0.7	2.10
		After 2hrs	9	2.75	0.4	1.14	4	3.05	0.6	1.28	11	2.53	0.7	1.98	9	3.02	1.0	3.24	8	3.10	1.1	3.19	16	2.30	0.9	3.20
		6 hrs	8	2.55	0.8	2.34	5	3.21	0.3	0.63	11	2.42	1.1	4.26	7	2.89	0.9	2.78	11	2.74	0.9	3.08	13	2.10	1.1	3.48
		1 day	8	3.35	1.6	5.24	9	2.90	0.7	2.27	13	2.28	0.7	3.04	12	2.66	0.7	2.67	9	3.20	0.8	2.57	17	2.51	0.9	3.27
		2 days	8	2.26	0.8	2.35	4	2.97	1.1	2.57	7	2.20	0.7	2.01	16	2.98	0.9	3.09	12	2.69	0.7	2.54	12	2.44	0.9	3.50
Period (s)	fast	Before	17	0.24	0.1	0.27	11	0.23	0.1	0.16	14	0.26	0.1	0.31	18	0.23	0.1	0.23	19	0.27	0.1	0.47	15	0.28	0.1	0.25
		After	16	0.23	0.1	0.27	14	0.22	0.1	0.22	11	0.21	0.0	0.15	11	0.23	0.1	0.26	8	0.26	0.1	0.32	9	0.39	0.3	0.86

		After 2hrs	15	0.22	0.1	0.27	17	0.21	0.0	0.12	12	0.24	0.1	0.18	13	0.21	0.0	0.11	14	0.25	0.1	0.28	7	0.30	0.1	0.30
		6 hrs	16	0.26	0.1	0.36	16	0.19	0.0	0.11	12	0.22	0.1	0.16	15	0.26	0.1	0.20	11	0.22	0.1	0.23	10	0.25	0.1	0.15
		1 day	13	0.29	0.1	0.39	8	0.20	0.0	0.13	10	0.22	0.1	0.20	10	0.25	0.1	0.19	13	0.21	0.1	0.21	6	0.29	0.1	0.28
		2 days	6	0.28	0.0	0.13	8	0.20	0.0	0.11	16	0.22	0.1	0.25	6	0.26	0.1	0.14	10	0.24	0.1	0.19	11	0.24	0.1	0.26
		Before	7	0.47	0.2	0.54	10	0.31	0.1	0.33	9	0.38	0.1	0.31	4	0.40	0.0	0.09	3	0.37	0.2	0.37	8	0.43	0.1	0.33
		After	8	0.41	0.2	0.51	7	0.34	0.1	0.28	12	0.49	0.3	0.88	11	0.52	0.2	0.61	14	0.56	0.3	1.10	14	0.68	0.2	0.78
	slow	After 2hrs	9	0.37	0.1	0.13	4	0.34	0.1	0.16	11	0.45	0.1	0.37	9	0.36	0.1	0.28	8	0.38	0.1	0.36	16	0.57	0.3	0.74
		6 hrs	8	0.42	0.1	0.34	5	0.32	0.0	0.06	11	0.49	0.2	0.61	7	0.42	0.2	0.56	11	0.42	0.2	0.61	13	0.62	0.3	1.08
		1 day	8	0.37	0.2	0.49	9	0.37	0.1	0.30	13	0.50	0.2	0.94	12	0.41	0.1	0.30	9	0.34	0.1	0.26	17	0.54	0.4	1.78
		2 days	8	0.51	0.2	0.60	4	0.38	0.1	0.31	7	0.54	0.2	0.54	16	0.38	0.1	0.31	12	0.41	0.1	0.37	12	0.47	0.2	0.61
		Before	15	5.95	1.5	4.36	10	6.80	1.3	4.10	14	6.56	0.8	3.10	18	6.42	1.1	4.54	19	6.27	1.2	5.28	15	6.00	1.1	4.59
		After	13	6.69	1.6	5.32	13	6.78	1.1	3.84	10	5.67	1.2	4.38	11	6.84	0.8	2.17	8	5.21	0.8	2.58	8	6.20	1.6	4.57
	fast	After 2hrs	14	6.42	1.0	3.79	15	6.70	0.7	3.08	12	5.78	0.7	2.52	12	6.86	1.2	4.18	13	5.77	1.7	5.89	7	7.11	1.5	4.25
		6 hrs	16	6.14	1.1	4.27	14	6.31	1.1	4.20	11	5.75	1.2	4.06	15	6.50	0.6	2.52	11	5.84	0.9	2.75	9	6.43	1.2	4.17
		1 day	13	6.06	1.2	4.51	8	5.47	0.9	2.55	10	5.82	1.3	4.00	10	5.76	0.7	2.64	13	5.70	1.5	5.57	6	5.51	0.5	1.30
Stride length (cm)		2 days	6	6.23	1.2	2.83	8	4.93	1.0	3.19	15	4.95	0.7	2.66	6	5.40	1.1	2.88	10	4.76	0.8	3.00	10	5.52	1.0	2.93
		Before	7	5.13	0.8	2.10	10	5.58	1.1	3.04	9	5.73	0.9	3.09	4	5.34	0.8	1.87	3	4.44	1.2	2.29	8	5.16	0.7	2.06
		After	8	5.66	1.1	3.03	7	5.34	1.2	3.88	12	5.08	1.0	3.55	11	4.97	0.6	1.70	14	4.07	0.9	2.62	12	4.18	1.0	3.60
	slow	After 2hrs	9	5.44	1.1	3.82	4	4.86	0.5	1.23	10	4.71	0.6	1.84	8	4.72	0.8	1.69	8	4.97	1.9	5.61	16	3.90	0.8	2.96
		6 hrs	7	5.63	0.7	1.89	5	4.65	0.7	1.47	11	4.63	0.7	2.03	7	4.70	0.6	1.55	11	5.31	1.6	4.70	13	4.67	1.2	3.94
		1 day	8	4.86	0.6	1.63	9	4.45	1.2	3.29	13	4.51	0.7	2.37	12	4.39	0.7	2.55	9	4.65	1.3	3.53	17	4.80	1.0	4.71
		2 days	8	4.22	0.9	3.04	2	4.35	1.2	1.66	7	4.02	0.9	2.66	16	3.62	0.5	1.77	12	4.17	1.0	3.48	12	4.03	1.3	4.09

Supplementary table 2

Size summary by sex of *Prionostemma* sp.1 harvestmen according to the assigned treatment. "X" marks the comparisons in which each treatment was used (see Methods). Overall legs 4 were longer in males than in females (Two-way ANOVA $F_{1/123}$=9.46, *P*=0.003). Even though we found that leg length differed between treatments ($F_{5/123}$=3.61, *P* = 0.004), we did not include it as factor in the GLMM (see Table S2) because post hoc comparisons showed that only the 2S and 2A were different (*P*=0.005), which were not included in the same statistical comparison (see Results). The leg 4 was longer for males than for females (*t*-test$_{153.29}$=-3.25, *P*=0.001). However, the variation in leg length did not affected the variation in locomotor variables, hence it was not included in the models (See Methods and Results).

Included in analysis			Treatment		Sample size (n)		leg 4 length (mm)	
A. Number legs lost	B. Type of legs lost	C. Side of body	Code	Description	Male	Female	Male	Female
x	x	x	C	Control	12	12	67.5 ± 0.9	64.1 ± 1.4
x			1L	Missing one locomotor leg	10	11	67.1 ± 1.6	64.7 ± 1.2
x	x	x	2L	Missing two locomotor legs (one on each side)	13	10	69.7 ± 1.3	66.0 ± 0.8
	x		2S	Missing 2 sensory legs	12	10	64.3 ± 1.9	63.5 ± 3.0
		x	2A	Missing 2 locomotor leg in the same side	17	5	70.9 ± 0.8	65.1 ± 0.6
x			3L	Missing 3 locomotor legs	10	13	68.1 ± 1.3	67.0 ± 1.2

Supplementary table 3

Results for the univariate tests on stride, postural and performance variables of *Prionostemma* sp.1 harvestmen moving through a horizontal track, according to different leg condition treatments. The number in the treatment represents the number of legs animals lost and the letter represents the type of lost (L: missing locomotor legs, S: missing sensory legs, and A: missing two locomotor legs in the same side of the body, hence, asymmetric loss). Table shows results of GLMs using time as predictor. Statistically significant results are marked in red.

Treatment	Analysis	Parameter	Duty Factor (prop.)	Stride frequency (Hz)	Period (s)	Stride length (cm)	3D sinuosity per time	Minimal height (cm)	Maximal height (cm)	Average horizontal velocity (cm/s)	Maximal horizontal acceleration (cm/s2)
**Control**	model	F	0.50	0.34	0.30	1.90	1.41	0.63	0.96	1.1	0.56
		df	2/63	2/63	2/63	2/58	2/56	2/58	2/63	2/66	2/58
		P	0.64	0.72	0.74	0.16	0.25	0.53	0.39	0.34	0.58
	Post hoc Tukeys	Before - Immed after	ns	ns	ns	ns	ns	ns	ns	ns	ns
		Bef – 2 days later	ns	ns	ns	ns	ns	ns	ns	ns	ns
		Immed after – 2 days later	ns	ns	ns	ns	ns	ns	ns	ns	ns
**1L**	model	F	3.45	0.41	0.47	6.17	2.37	2.41	0.47	2.58	0.08
		df	2/56	2/56	2/56	2/54	2/52	2/52	2/55	2/55	2/49
		P	0.04	0.67	0.63	0.004	0.10	0.10	0.63	0.09	0.92
		Bef - Imm after	0.03	ns	ns	0.97	ns	ns	ns	ns	ns

	Post hoc Tukeys	Bef – 2 days	0.2	ns	ns	0.01	ns	ns	ns	ns	ns
		Immed after – 2 days	0.75	ns	ns	0.006	ns	ns	ns	ns	ns
2L	model	F	18.9	0.2	0.73	7.34	0.63	3.64	0.97	1.04	0.14
		df	2/66	2/66	2/66	2/65	2/65	2/65	2/66	2/66	2/65
		P	0.001	0.81	0.49	0.001	0.51	0.03	0.39	0.36	0.87
	Post hoc Tukeys	Before - Immed after	<0.001	ns	ns	0.02	ns	0.02	ns	ns	ns
		Bef – 2 days	<0.001	ns	ns	0.001	ns	0.3	ns	ns	ns
		Immed after – 2 days	0.99	ns	ns	0.67	ns	0.47	ns	ns	ns
2S	model	F	0.03	2.02	3.31	7.21	1.68	8.93	0.54	4.72	2.02
		df	2/63	2/63	2/63	2/63	2/62	2/63	2/63	2/63	2/62
		P	0.97	0.14	0.04	0.015	0.2	0.004	0.58	0.01	0.14
	Post hoc Tukeys	Bef - Imm after	ns	ns	0.04	0.66	ns	0.002	ns	0.11	ns
		Bef – 2 days	ns	ns	0.21	0.001	ns	<0.001	ns	0.01	ns
		Immed after – 2 days	ns	ns	0.68	0.02	ns	0.95	ns	0.59	ns
2A	model	F	11.5	4.44	6.12	7.69	6.12	25.7	8.06	7.58	4.91
		df	2/63	2/63	2/63	2/63	2/63	2/62	2/63	2/63	2/59
		P	<0.001	0.016	0.004	0.001	0.004	<0.001	0.001	0.001	0.01
	Post hoc Tukeys	Before - Immed after	<0.001	0.09	0.015	<0.001	0.04	<0.001	<0.001	0.0015	0.007
		Bef – 2 days	0.01	0.72	0.95	0.13	0.62	<0.001	0.13	0.01	0.29

		Immed after – 2 days	0.19	0.015	0.007	0.12	0.03	0.11	0.1	0.81	0.23
3L	model	F	7.6	4.94	4.19	2.65	2.47	5.03	1.68	6.32	4.86
		df	2/62	2/66	2/66	2/63	2/64	2/63	2/64	2/64	2/59
		P	0.001	0.001	0.02	0.08	0.10	0.01	0.2	0.003	0.01
	Post hoc Tukeys	Bef - Imm after	0.001	0.007	0.015	ns	ns	0.36	ns	0.01	0.008
		Bef – 2 days	0.01	0.18	0.18	ns	ns	0.006	ns	0.007	0.27
		Immed after – 2 days	0.73	0.36	0.52	ns	ns	0.2	ns	0.99	0.28
